# DNA Damage Repair Genes Controlling Human Papillomavirus (HPV) Episome Levels under Conditions of Stability and Extreme Instability

**DOI:** 10.1371/journal.pone.0075406

**Published:** 2013-10-02

**Authors:** Terri G. Edwards, Thomas J. Vidmar, Kevin Koeller, James K. Bashkin, Chris Fisher

**Affiliations:** 1 NanoVir, Kalamazoo, Michigan, United States of America; 2 BioStat Consultants, Portage, Michigan, United States of America; 3 Department of Chemistry & Biochemistry, University of Missouri-St. Louis, St. Louis, Missouri, United States of America; International Centre for Genetic Engineering and Biotechnology, Italy

## Abstract

DNA damage response (DDR) genes and pathways controlling the stability of HPV episomal DNA are reported here. We set out to understand the mechanism by which a DNA-binding, N-methylpyrrole-imidazole hairpin polyamide (PA25) acts to cause the dramatic loss of HPV DNA from cells. Southern blots revealed that PA25 alters HPV episomes within 5 hours of treatment. Gene expression arrays identified numerous DDR genes that were specifically altered in HPV16 episome-containing cells (W12E) by PA25, but not in HPV-negative (C33A) cells or in cells with integrated HPV16 (SiHa). A siRNA screen of 240 DDR genes was then conducted to identify enhancers and repressors of PA25 activity. Serendipitously, the screen also identified many novel genes, such as TDP1 and TDP2, regulating normal HPV episome stability. MRN and 9-1-1 complexes emerged as important for PA25-mediated episome destruction and were selected for follow-up studies. Mre11, along with other homologous recombination and dsDNA break repair genes, was among the highly significant PA25 repressors. The Mre11 inhibitor Mirin was found to sensitize HPV episomes to PA25 resulting in a ∼5-fold reduction of the PA25 IC50. A novel assay that couples end-labeling of DNA to Q-PCR showed that PA25 causes strand breaks within HPV DNA, and that Mirin greatly enhances this activity. The 9-1-1 complex member Rad9, a representative PA25 enhancer, was transiently phosphorylated in response to PA25 treatment suggesting that it has a role in detecting and signaling episome damage by PA25 to the cell. These results establish that DNA-targeted compounds enter cells and specifically target the HPV episome. This action leads to the activation of numerous DDR pathways and the massive elimination of episomal DNA from cells. Our findings demonstrate that viral episomes can be targeted for elimination from cells by minor groove binding agents, and implicate DDR pathways as important mediators of this process.

## Introduction

Human papillomavirus (HPV) infection results in establishment of the viral genome as a circular, multi-copy, extrachromosomal double-stranded DNA (dsDNA), or episome, within the proliferating cell compartment of stratified squamous epithelia [1]. Persistent HPV infection, defined as the length of time that HPV DNA is detectable following an initial positive clinical test, is considered the greatest risk factor for HPV-dependent carcinogenic progression [2], [3], [4]. Controversy exists over what constitutes or abets a persistent infection (see [3] for discussion), but factors such as status of host immune system and viral immune evasion appear to be key [3], [5], [6], [7]. Antiviral therapies for HPV remain an important, unmet medical need, but have not been developed for a variety of reasons including the small HPV genome, which encodes few traditional antiviral targets. Therefore, alternative approaches to antiviral therapies are important. To this end, a series of DNA-binding, N-methylpyrrole-imidazole hairpin polyamides (PAs) that target HPV episome DNA for elimination from cells has been described [8], [9]. These compounds trigger massive viral DNA instability and rapid loss from cells by an unknown mechanism.

HPV must evade innate cellular defense mechanisms to be maintained in cells [7], [10], [11]. DNA damage response (DDR) pathways are increasingly recognized as a central host defense mechanism that must be subverted, and are often utilized, by DNA viruses to establish a persistent infection [9], [12], [13]. DNA viruses have a complex relationship with DDR pathways [12]. Foreign DNA activates the DDR, which protects the host cell genome. Conversely, viruses counteract and often exploit the DDR to promote their survival and life cycle. The ataxia-telangiectasia mutated (ATM) and ATM and Rad3-related (ATR) serine/threonine protein kinases are important sensors of DNA damage. ATM responds to dsDNA breaks (DSBs), while ATR senses a variety of DNA insults such as stalled replication forks and ssDNA exposure and harm [14]. ATM and/or ATR signaling is activated by most DNA viruses due to recognition of viral genomes as damaged DNA, in response to replication stress, or by viral activation of these pathways to promote facets of its life cycle [15]. Cells carrying HPV episomes show constitutive activation of DDR elements including ATR, ATM, Chk1, Chk2, BRCA1, and Nbs1 [9], [16], [17], and inhibition of the ATR/Chk1 pathway results in loss of HPV episomes from cells [9]. HPV-encoded proteins have also been shown to directly activate DDR pathways. HPV E1, the viral helicase that licenses HPV replication, activates ATM and ATR while causing dsDNA breaks (DSBs), which are impaired by E2 [18], [19]. HPV E7 binds ATM and promotes Chk2 regulated caspase-dependent activation of HPV E1, and pharmacological inhibition of ATM impedes productive HPV replication in differentiating keratinocytes [16].

Other DDR response pathways have also been implicated in HPV infection. Fanconi Anemia (FA) is a genome instability syndrome causing extremely high rates of squamous cell carcinoma (SCC). FA is caused by mutation of 1 of the 15 members of the FA pathway, which assemble in the nucleus to form a large ubiquitin ligase important in DNA repair [20]. While there are conflicting reports on the role of HPV in SCC in FA patients [21], [22], the intact FA pathway appears to function as an HPV suppressor in laboratory studies [23], [24]. Homologous recombination (HR) and non-homologous end-joining (NHEJ) are the two primary processes of dsDNA break repair in mammalian cells. The Mre11-Rad50-Nbs1 (MRN) complex plays a role in both HR and NHEJ as a dsDNA break sensor [25], [26]. CtIP, a physical and functional partner of the MRN complex, is also required for efficient HR and is essential for dsDNA break resection [27]. The DNA viruses SV40 and EBV have been shown to use HR for efficient replication [15], and multiple members of the HR pathway are recruited to HPV nuclear foci where they play a role in productive replication and, possibly, in HPV episome maintenance [16], [28]. Our own studies demonstrated that ATM knockdown by siRNA, but not pharmacological inhibition, results in significant HPV episome loss from cells suggesting a structural, rather than enzymatic, role in HPV episome maintenance [9].

Understanding the mechanism by which antiviral PAs destabilize and eliminate HPV episomes from cells may shed light upon viral DNA evasion of innate immunity and persistence in cells [8], [9]. The degree and time course of HPV episome loss suggests that PAs trigger active elimination of viral DNA by the cell. Smaller polyamides and other minor groove-binding agents are known to affect DNA structure in a number of quantitative and qualitative ways [29], [30], [31], [32]. For example, 6- and 8-ring polyamides, significantly smaller than PA25, were shown by gel shift assays of ligation ladders to affect DNA bending in a manner dependent on DNA and polyamide sequence. The consequences of binding of larger PAs (such as PA1, PA25, and other anti-HPV compounds which bind to a minimum of ∼1 helical turn) for DNA structure and conformation are unknown.

Here we show that PA25 causes HPV-specific alterations in expression of numerous DDR genes. A siRNA screen targeting 240 DDR genes was then utilized to identify repressors and enhancers of PA25 antiviral activity with a high degree of statistical confidence. The 9-1-1 and MRN complexes stood out as significant contributors to PA25 activity based upon results from the siRNA screen and gene expression studies, and were selected for follow up studies. PA25 was found to trigger phosphorylation of 9-1-1 complex member Rad9 in a time- and HPV-dependent manner. PA25 is also shown to cause dsDNA breaks within the HPV genome, and this activity is enhanced by Mirin, an inhibitor of Mre11 endonuclease activity, which sensitizes HPV episomes to PA25. Together our findings show that PA25 causes structural alterations and DSBs within HPV episomes resulting in activation of DDR pathways which are rate limiting for episome loss. These findings contribute to understanding how DDR pathways control the massive instability of HPV episomes in the presence of a novel DNA-targeted compound, and suggest that these pathways regulate the initiation of a poorly understood process leading to viral DNA destruction.

## Materials and Methods

### Cells and Cell Culture

HPV-maintaining human keratinocytes were maintained and passaged as previously described [8], [33]. C33A and SiHa cells (ATCC, Rockville, MD) were cultured in Dulbecco's Modified Eagle's Media containing 10% fetal bovine serum (Invitrogen). Fluorescence activated cell sorting (FACS) was conducted as previously described [9].

### Quantification of HPV Episome Levels

HPV episome copy number was tracked via Q-PCR using L1-specific primers and TaqMan® probe as previously described [8], [9], [33]. IC50 values were calculated by non-linear regression using XLFIT (IDBS). For drug treatment, W12E cells were pre-treated with 100 µM Mirin [34] (Sigma, Cat # M9948) or 0.1% DMSO for 24h. Media was removed and fresh media containing the indicated doses of PA25 or 0.1% DMSO was added, and cells incubated an additional 24h. Total DNA was harvested using DNAzol (Invitrogen, Cat # 10503-027) according to manufacturer’s recommendation, and 20 ng total DNA analyzed as above by Q-PCR.

### Polyamide Preparation

Polyamides 11 (PA11) and 25 (PA25) were prepared by solid phase, Boc-protected peptide methodology as reported [35]. Purification was carried out as described in the same literature report, using reverse-phase HPLC with 0.1% trifluoroacetic acid in the mobile phase. Analytical HPLC/mass spectrometry showed high purity. The exact mass of compound 25 was reported previously to help characterize its composition [8]. PA11 was also reported previously by another group [36], but no characterization data were given. Therefore, we provide the PA11 High Resolution Mass Spectral data here: C_58_H_71_N_21_O_10_ M^+^ (theoretical) 1221.5684 M^+^ (measured, ESI HRMS) 1221.5707. Detailed chemical characterization of both compounds, for example by 600 MHz ^1^H and ^13^C NMR, will be reported in a more chemical journal as part of the characterization of our entire library of active and inactive polyamides.

### Southern Blotting

T75 flasks of W12E cells were treated with 1 µM PA25 or 0.1% DMSO for the indicated times. Total cellular DNA was extracted by lysing the cells with 20 mM Tris-HCL (pH 8), 100 mM EDTA, 150 mM sodium chloride, and 1% sodium dodecyl sulfate (SDS) containing 50 ug/ml Proteinase K (Invitrogen, Cat # 100005393) and incubated overnight at 37°C. Samples were extracted with phenol:chloroform:isoamyl alcohol (25:24:1, v/v) until interphase was clear followed by 2X chloroform extraction. Total DNA was precipitated with 2 volumes of ethanol and incubated overnight at −20°C. Pellets were re-suspended in 0.5 mL Tris-EDTA (TE) buffer (PH 8.0), sheared by passing through an 18-gauge needle and 50 ug/ml RNAse A (Sigma R4642) added for 1h at 37°C. DNA was again phenol:chloroform extracted and ethanol precipitated. DNA pellets were re-suspended in TE buffer and 5 ug was digested with BamHI or HindIII overnight. DNA was electrophoresed in the presence of 0.5 ug/mL ethidium bromide at 5 V/cm for 18h, transferred onto positively-charged nylon membranes and probed with full-length HPV16 genomic DNA (random-primed with [^32^P]dCTP). Membranes were exposed to phosphor screens and imaged with a Molecular Dynamics Phosphorimager.

### Gene Expression Arrays and RT-PCR

W12E cells were seeded onto 6-well plates (approx. 100,000 cells) and the following day treated with 10 uM PA25, 10 uM control PA11, or 0.1% DMSO in W12E cell growth media. RNA was extracted 48hr later using the RT^2^ qPCR-Grade RNA Isolation Kit (SABiosciences, Cat # PA-001) and quality confirmed by agarose gel assessment of 18S/28S ribosomal RNA. RNA from each treatment group was reverse transcribed using Maxima First Strand cDNA Synthesis Kit (Fermentas, Cat # K1641). Drug effects on gene expression were analyzed for the following pathways: Human Apoptosis (Cat # HPA-I), Human DNA Repair I & II (Cat # HDRL-I and HDRL-II), and Human Cell Cycle (Cat # HCC-I). All PCR Arrays were obtained from RealTimePrimers. Primers were supplied lyophilized in 96-well plates with each pathway array containing unique gene-specific primer pairs for 88 target genes (TG). An additional eight primer pairs were supplied for 8 control genes. Primer stocks were re-suspended at 10 µM and added to PCR reactions at [100 nM] final along with Sybr Green Master Mix (Fermentas, Cat # K0221) and 2.5 ng cDNA per well of 96-well plates. Q-PCR was performed using the following cycling conditions: 1X 95°C for 10 min followed by 40 cycles of 95°C for 10 sec, 58°C for 45 sec. Three independent experiments were performed for each array, and the ΔCt calculated for each target gene (TG) against the average of all control genes (CG) as follows (ΔCt  =  Ct (CG) – Ct (TG). This approach yielded 9 pair-wise comparisons for each gene (3 DMSO-treated ΔCt values X 3 PA-treated gene ΔCt values). Genes were scored as affected by PA if there was a two-fold or greater change (2-fold change  =  1 ΔΔCt value) in gene expression compared to DMSO-treated cells in ≥ 7/9 pair-wise comparisons.

### siRNA Screen

Targeted siRNAs were purchased from Dharmacon (Dharmacon ON-TARGETplusRTF® SMARTpool® siRNA Library - Human DNA Damage Response; H-106005, Lot 11152) and supplied lyophilized on 96-well tissue-culture plates. A total of 240 genes were provided on three separate 96-well plates, with each plate containing 80 unique genes as well as non-targeting (NT) and cyclophilin-targeting control siRNAs. Each of the 4 independent experiments was conducted against all 240 genes along with controls. Transfection reagent (DharmaFECT1; Dharmacon cat# T-2001-02) was diluted to a final 0.15 µL per well with Dharmacon Cell Culture Reagent (cat# B-004500-100). 25 µL was added to each well and incubated 30 min at room temperature to fully re-suspend siRNAs. W12E or HPV31 maintaining cells were plated at 4500 cells/well in 100 µL complete E media (no antibiotic) with J2 3T3 feeder layer. Cells were incubated 72hr with siRNAs, media was changed to include 0.1% DMSO or 1 µM PA25 in 100 µL complete E media (no antibiotic), and then cells incubated an additional 24hr. At the end of the treatment period, cells were lysed and DNA harvested using a Wizard SV 96 Genomic DNA Purification Kit (Promega #A2370). siRNA and PA25 effects on cellular HPV episome levels was measured via TaqMan® PCR using HPV16 or HPV31-specific L1 primers as previously described [8], [9], [33].

Statistical analysis of the siRNA screen is described in detail in Supporting Information (Statistical Methods S1 in File S1, Figures S3 and S4 in File S1, and Table S2 in File S1). Briefly, two sets of data were generated for each of the 4 experiments. Set 1 examined the effects of 240 siRNAs on HPV episome levels, while set 2 looked at the effects of siRNAs on HPV episome levels after treatment with PA25. Set 1 positive hits were defined as siRNAs that gave a ≥2-fold change in HPV episome copy number in ≥5 out of 6 pair-wise comparisons in at least 3 out of 4 independent experiments when compared with the 6 control values on each day. Set 2 positive hits, which represent gene effects on PA25 antiviral activity, were identified as those siRNAs that resulted in a ≥2-fold difference in episome copy number as determined from the ΔΔCt (Set 2 value - Set 1 value) for any given siRNA. Gene hits for Set 2 also needed to be significant in at least 3 out of 4 independent experiments. The statistical methods employed are presented in greater detail in Supporting Information (Statistical Methods S1 in File S1, Figures S3 and S4 in File S1, and Table S2 in File S1). A list of all 240 genes employed in the siRNA screen is provided in Table S3 in File S1.

### Validation of Gene Hits

Genes were confirmed if at least 2 out of the 4 individual siRNA sequences recapitulated the data from the initial screen. Gene hits were verified by de-replication of Dharmacon SmartPool siRNAs into the 4 individual sequences. Singular siRNAs were reverse transfected into W12E cells at a final concentration of 50 nM, and then cultured for 72h at which time media was changed and either 1 µM PA25 or 0.1% DMSO was added. Cells were cultured an additional 24h, DNA harvested, and Q-PCR performed as described for the initial screen.

### Gene-specific siRNA knockdown and off-target effects

A subset of siRNAs represented in the siRNA screen was randomly chosen to analyze for specific siRNA knockdown and determination of potential off-target effects by reverse transcription PCR (RT-PCR). W12E cells were split onto 6-well plates and transfected 48h later with 50 nM siRNA (Dharmacon) using 3 µL transfection reagent per well in a total volume of 2 mL/well. Cells were cultured for 72h with siRNAs at which time RNA was extracted and reverse transcribed using Maxima First Strand cDNA Synthesis Kit (Fermentas, Cat #K164125). 2.5 ng of cDNA was used per Q-PCR reaction (SYBR® Green; Roche) using PCR primer sets obtained from IDT (see Table S4 in File S1) at a final concentration of 300 nM. All 22 primer sets were run against each siRNA transfection in a matrix to validate siRNA specificity and potential off-target effects. PCR conditions were as follows: 10 min 95°C, 40 cycles at 95°C 10 sec, 60°C 10 sec, 72°C 10 sec.

### Western Blotting

T75 flasks of W12E and C33A cells were treated with 1 µM PA25 and harvested at different times. Cells were trypsinized, washed with ice-cold phosphate-buffered saline (PBS), and lysed in radioimmunoprecipitation assay (RIPA) buffer (25 mM Tris-HCL pH 7.5, 150 mM NaCl, 1% NP-40, 2% sodium deoxycholate, 0.1% SDS) supplemented with 2X Halt Protease & Phosphatase Inhibitor Cocktail (Thermoscientific, catalog no. 1861284). Lysates were incubated with 150 U/mL DNAse I (Thermoscientific, catalog no. 89835) for 30 minutes at room temperature with mixing. Protein concentration was determined by BCA Assay (Thermoscientific, catalog no. 23227) and 50 µg protein separated on Tris-Glycine 4−20% gels (NuPAGE). Gels were transferred onto PVDF membranes with an iBLOT system (Invitrogen), and membranes blocked in 5% milk/TBST (20 mM Tris-HCL pH 7.5, 150 mM NaCl, 0.1% Tween 20) overnight at 4°C. Blots were incubated at room temperature for 2hr with phospho-Rad9 antibody (1∶200; Santa Cruz, sc-130213) or pan-Rad9 antibody (1∶200; Santa Cruz, sc-32489) and detected with goat anti-rabbit poly-HRP secondary antibody (1∶25,000; Pierce, Cat # 32260) and donkey anti-goat IgG-HRP secondary antibody (1∶25,000; Santa Cruz, sc-2020), respectively.

### Detection of HPV DNA Breaks by End-labeling Coupled to Q-PCR (ELCQ)

W12E cells were pre-treated with 0.1% DMSO or 100 uM Mirin for 24h followed by treatment with 0.1% DMSO or 1 uM PA25 for 5h. HPV16 episomal DNA was isolated from W12E cells by standard alkaline lysis plasmid preparation procedures [37]. Following neutralization, the supernatant was cleared by centrifugation and DNA precipitated by addition of 0.7X volume isopropanol. DNA pellets were washed 3X with 70% ethanol and resuspended in TE buffer (pH 8.0). Terminal deoxynucleotidyl transferase (TdT) tailing reactions were conducted in the presence of Biotin-16-dUTP as follows: 20 uL reactions containing 1 ug DNA, 2 uL nucleotide mix (35 uM Biotin-16-dUTP final, Roche, Cat # 11093070910), 4 uL [25 mM] CoCl_2_ (5 mM final), 4 uL 5X TdT buffer, and 1 uL TdT enzyme (400U, Roche, Cat # 03333566001) were incubated for 30 min at 37°C. For each DNA sample, a negative control reaction was set up containing all reaction components except TdT. DNA was isolated from unincorporated nucleotides by ethanol precipitation. Biotin-Streptavidin pull-down assays were subsequently conducted as follows: 50 uL of Streptavidin-Sepharose 4B Conjugate beads (Invitrogen, Cat # 43-4341) were used for each pull-down; to block non-specific binding, Sepharose beads were pre-incubated in 500 uL binding/wash buffer (BWB, 20 mM Tris-HCL pH 8.0, 5 mM EDTA, 2 M NaCl, 0.1% NP40) containing 100 ug salmon sperm DNA and 100 ug purified BSA for 1h at RT with shaking; biotin end-labeled DNA was resuspended in 100 uL BWB containing 100 ug of both salmon sperm DNA and BSA, added to pre-blocked Sepharose beads and incubated 30 min at RT. Beads were washed 5X with BWB and DNA eluted by resuspending beads in 25 uL 95% formamide, 10 mM EDTA pH 8.0 and incubating at 65°C for 10’. Eluted DNA was then diluted 10-fold with water. HPV DNA copy number was quantified via TaqMan real-time PCR (described above). Specific pull-down of tailed HPV16 was calculated by subtracting total copies pulled down in the absence of TdT (non-specific) from total copies pulled down in the TdT-biotin tailing reaction (specific).

## Results

### Compounds that Destabilize HPV Episomes

**Figure 1:**
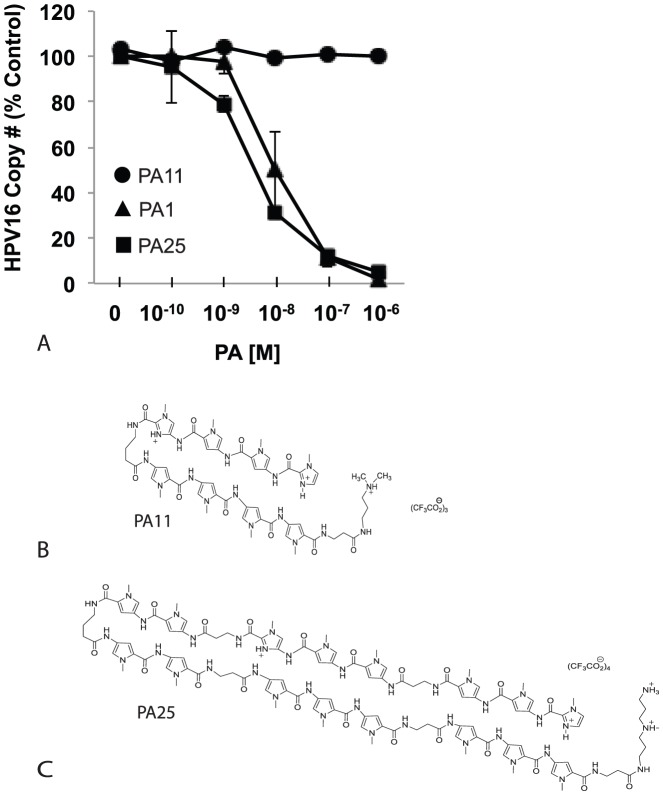
Antiviral activity and structure of anti-HPV N-methylpyrrole-imidazole polyamides following 48 hours of treatment in W12E cells. A. PA1 and PA25 dramatically decrease HPV16 episome levels in W12E cells while the related PA11 has no effect. B. Structure of PA11. C. Structure of PA25.

**Figure 2:**
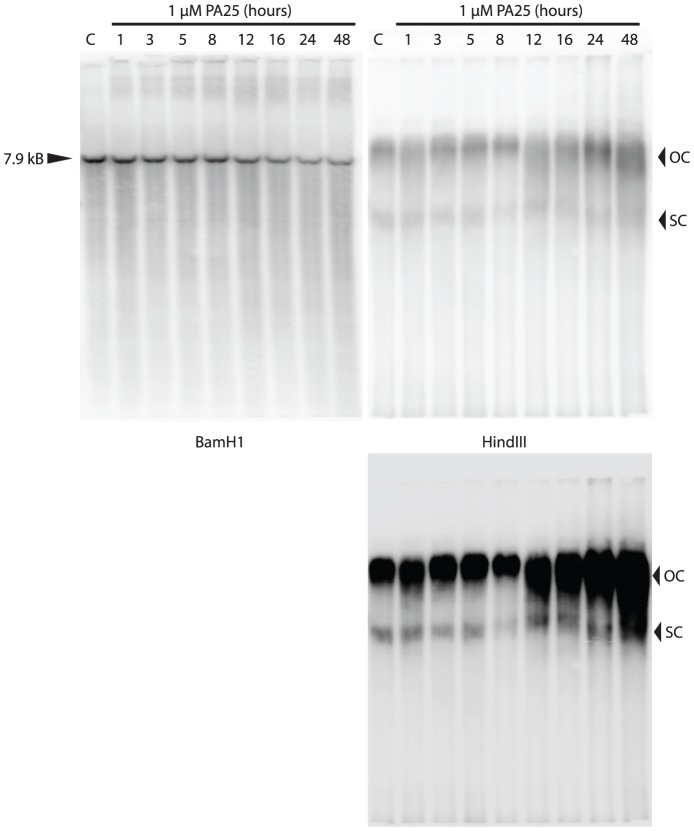
Southern blots of linearized (left) and intact (right) HPV16 episomes over time following treatment with 1 µM PA25 for 48 hours. The blots are loaded identically except HPV16 was linearized by BamH1 in one set of samples (left) or digested with HindIII, which does not restrict viral DNA (right). An additional, over-exposed HindIII blot is also provided. OC: open circle; SC: super-coiled.

PA1 and PA25 potently reduced HPV episome copy number in W12E cells while a related compound, PA11, had no effect (Figure 1A). The structure of PA25 and PA11 are shown (Figure 1B and 1C); the structure of PA1 was previously published [8]. Southern blots were conducted to examine the effects of PA25 on HPV DNA over 48 h. of treatment (Figure 2). The blots showed good agreement with compound Q-PCR potency data results (Figure 1A) with a time-dependent decrease in the BamH1 linearized episomal DNA observed. The corresponding blots of uncut HPV DNA (HindIII) did not show the clear quantitative decrease in viral DNA that was exhibited by the blots of linearized samples and Q-PCR, but PA25-dependant, qualitative changes to the OC and SC episome forms were noted in the later stages of the time course (Figure 2). PA25 (1 µM) caused both forms to migrate aberrantly and diffusely, a change that was particularly apparent after 8 h. of treatment. The lack of quantitative signal was attributed to increased hybridization of probe with the smeared viral DNA at later time points. Once the HPV episomal DNA was linearized with BamH1, all bands collapsed into a single band on a blot that was highly quantitative and correlated well with Q-PCR data (Figure 2). No evidence of viral DNA integration is seen in either blot, consistent with previous findings [8], [9].

### PCR Array Studies of PA25 Effects on Gene Expression

**Figure 3:**
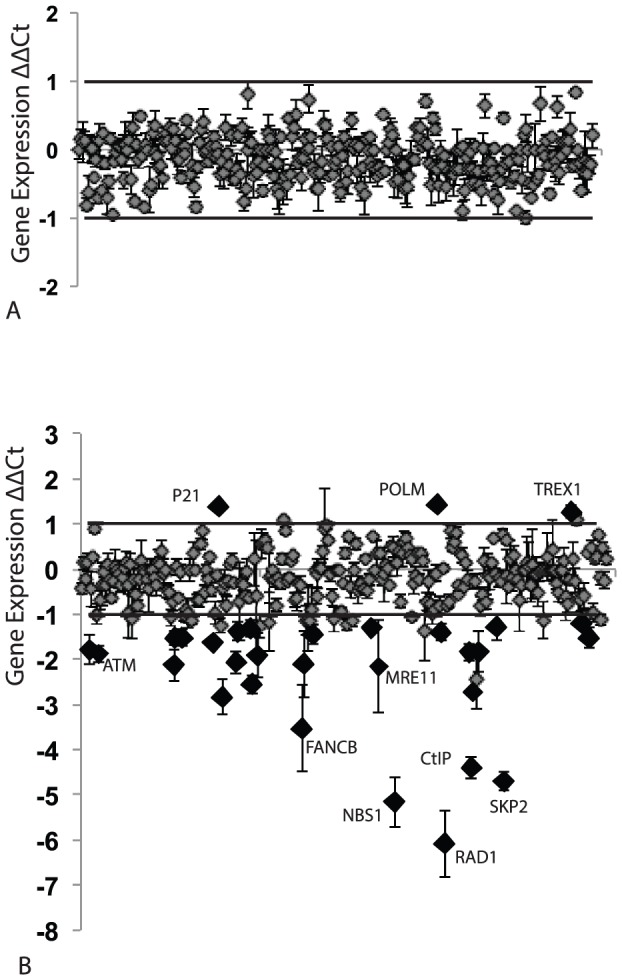
Effects of PA11 and PA25 on expression of cell cycle, apoptosis, and DDR genes in W12E cells. A. PA11, an inactive polyamide, does not significantly alter gene expression in W12E cells. B. PA25 significantly alters the expression of numerous genes in W12E cells. The expression of most genes is decreased in response to PA25 while 3 are significantly increased.

These results showed that the HPV episome was destabilized and lost following PA25 treatment, and suggested that it might be physically altered by PA25. A series of PCR array experiments were next conducted with HPV16-maintaining W12E cells in order to explore the effects of PA25 on gene expression. PA11 was run in these experiments as a control compound. PCR arrays covering apoptotic, cell cycle, and DDR pathways were selected for these initial experiments. PA11 did not significantly affect the expression of any genes according to our criteria: no genes gave a ΔΔCt of ≥1 in ≥7 of 9 pair-wise comparisons (Figure 3A). On the other hand, PA25 significantly altered the expression of numerous genes most of which exhibited decreased expression and were members of DDR or cell cycle pathways (Figure 3B and Table 1). RAD1 and NBS1 gave the greatest ΔΔCt values signifying down-regulation of these genes by 67- and 35-fold respectively (Figure 3B and Table 1). CtIP (RBBP8) was also among the most affected genes (21-fold down regulation). It was of high interest that MRE11 was also significantly down-regulated since it partners with NBS1 in the MRN complex, and in conjunction with CtIP promotes end resection during DSB repair (Figure 3B and Table 1). Three members of the Fanconi Anemia pathway (FANCB, FANCC, and FANCL) were also down regulated (Table 1). Only 3 genes showed statistically significant, increased expression, albeit marginal, in response to PA25: p21 (CDKN1A), POLM, and TREX1. A summary of all genes exhibiting significantly altered expression in the presence of PA25 is provided in Table 1.

**Table 1:** Genes whose expression is significantly altered by PA25 in W12E cells.

Gene	mRNA (Fold Δ)	Function/Pathway	
CDKN1A	2.62	CDK inhibitor 1A (p21)	**Cell Cycle**
CDKN1B	−2.36	CDK inhibitor 1B (p27, Kip1)	
CDKN2A	−7.16	CDK inhibitor 2A (p16)	
CCNB2	−4.38	G2/mitotic-specific cyclin-B2	
CCNC	−2.87	Cyclin C; regulates RNA polymerase II	
CCNE2	−2.87	G1/S-specific cyclin-E2	
CDK6	−3.10	promotes G1/S transition	
ANAPC4	−3.44	APC subunit 4	
RBL1	−6.57	retinoblastoma-like 1 (p107)	
RB1	−3.59	pRb	
ATM	−3.68	PI-3 kinase; DSB repair; HR	**DNA Damage Repair**
CHK2	−2.62	DDR ATM checkpoint effector	
CHK1	−4.17	DDR ATR checkpoint effector	
MRE11A	−4.47	MRN complex involved in DSB repair; HR	
NBS1	−35.70	MRN complex involved in DSB repair; HR	
CtIP (RBBP8)	−21.01	endonuclease; cooperates with MRN complex; HR	
RAD1	−67.65	9-1-1 complex member; exonuclease; BER	
XRCC4	−2.19	dsDNA break repair; NHEJ	
FANCB	−11.55	Fanconi anemia pathway	
FANCC	−4.32	Fanconi anemia pathway	
FANCL	−2.72	Fanconi anemia pathway	
CUL2	−2.49	E3 ubiquitin-conjugating complex member	
CUL3	−5.88	E3 ubiquitin-conjugating complex member	
UBE2N	−2.30	E2 ubiquitin-conjugating enzyme E2N	
SKP2	−25.93	SCF member; E3 ligase; p27, E7 and E6 degradation	
POLM	2.70	gap-filling polymerase; NHEJ	
POLQ	−2.63	DNA pol theta; interstrand crosslink repair; Alt-NHEJ	
TREX1	2.41	3' repair exonuclease 1	
DCLRE1A	−3.77	DNA cross-link repair 1A	
DCLRE1B	−2.39	protection of telomeres against NHEJ	
RECQL	−3.57	DNA helicase	
WRN	−2.89	DNA helicase, RecQ-like type 3	
RDM1	−5.37	RAD52 motif-containing protein 1	
MLH3	−2.47	mutL homolog, MMR	
LIG3	−2.31	DNA ligase; BER	
RPA4	−2.44	rep. protein A4; DSB repair	

**DSB:** double-strand break; **BER:** base excision repair; **MMR:** mismatch repair; **HR:** homologous recombination; **TLS:** translesion repair; **MMR:** mismatch repair; **NHEJ**: non-homologous end-joining.

To determine if changes in gene expression were due to PA25 interaction with HPV episomes, the same screen was conducted in the HPV-negative C33A cervical cancer cell line and in the HPV16 positive SiHa cell line. In both cases, the expression of only 7 genes was altered by PA25 treatment (Figure S1 and Table S1 in File S1), and only one of these genes (LIG3 in C33A cells) was among those altered in the HPV16-positive W12E cells. The genes altered in both C33A and SiHa cells showed remarkable similarity with only one gene difference for each cell type (Figure S1 and Table S1 in File S1).

Since a number of cell cycle genes exhibited altered expression in the presence of PA25 (Table 1), the effects of 10 µM PA25 on cell cycle progression was next examined. FACs analysis suggested that W12E cell progression through G2/M and S phase were slowed by PA25 resulting in small increases of cells in these phases with a concomitant decrease of the G0/G1 population (Figure S2 in File S1). However, these effects do not significantly alter cell growth since PA25, or other PAs, have not been found to effect cell numbers in cell viability assays [8].

### A siRNA Screen for DDR Genes that Modify HPV Episome Stability

**Figure 4:**
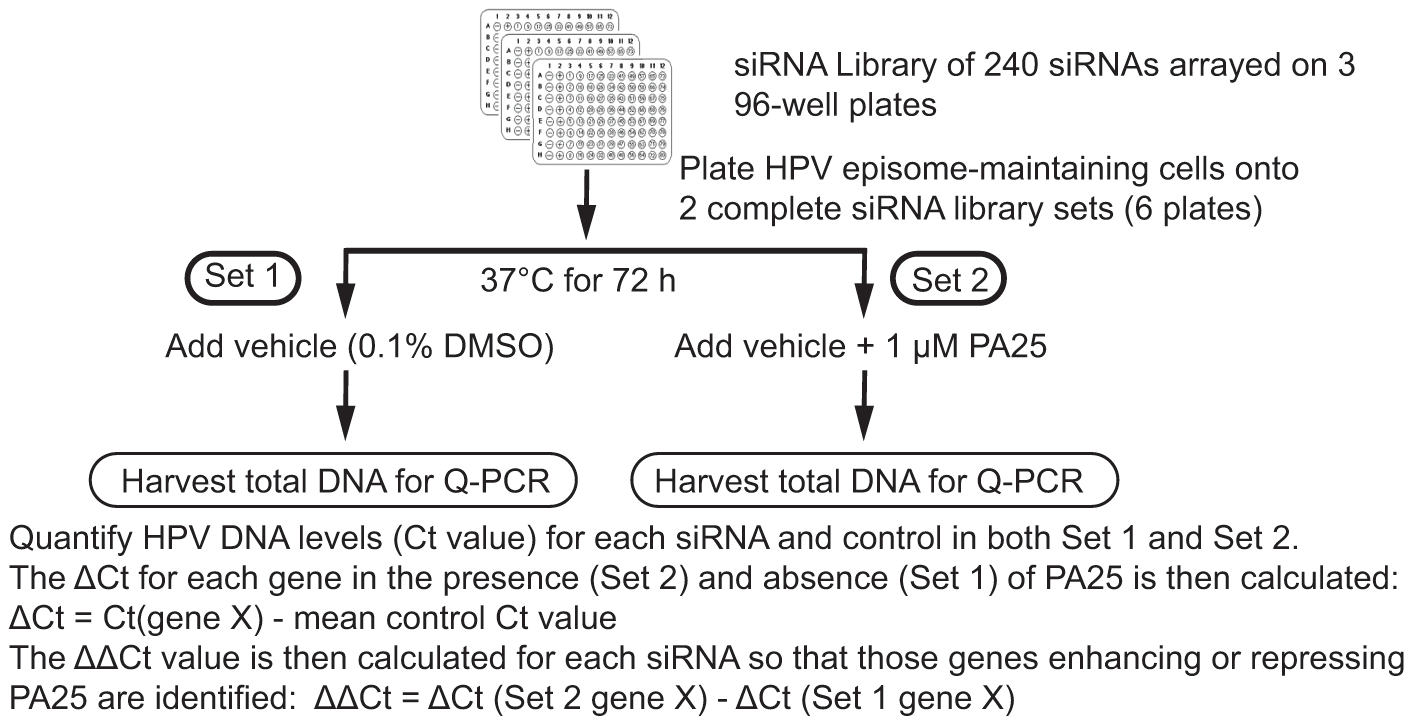
Experimental design of siRNA screen. A total of 4 experiments were conducted on 4 separate days with cells that were treated with either vehicle (Set 1) or vehicle plus PA25 (Set 2). Cells maintaining HPV16 (days 1−3) or HPV31 (day 4) were used in these experiments.

These results indicated that DDR pathways are triggered by PA25 and might play a role in its antiviral activity. With this as rationalization, a siRNA screen was undertaken to examine the role of 240 DDR genes in episome stability, and in HPV episome instability triggered by PA25. The overall screen design included 4 complete experiments: the first three experiments focused on HPV16 episome fate in W12E cells, while the final experiment examined HPV31 episomes in HPV31-containing cells (Figure 4). These experimental replicates were necessary to allow the identification of significant changes in episome copy number with a high degree of statistical confidence, and to assess episome stability for two HPV genotypes in different cellular backgrounds. Each experiment provided ΔCt values for HPV episomes in the presence of control siRNAs and 240 DDR siRNAs for cells receiving either vehicle (0.1% DMSO; Set 1) or 1 µM PA25 (Set 2). Thus, the data provided the opportunity to assess the effects of DDR siRNAs on HPV episome levels under conditions of stable maintenance (0.1% DMSO vehicle) or under conditions of massive instability (1 µM PA25) (Figure 4).

An evaluation of variability among siRNA control and experimental values assessed the degree of reproducibility of the screen between experiments, which were conducted on separate days. Popular measures such as the Z-factor that provides an appraisal of data quality [38] were not possible since no positive controls were available for use in the study. Therefore calculation of the coefficient of variation (CV) was performed for each experimental day for the control data. The CVs were all found to be well below 5% and fairly consistent across all experimental days indicating a high degree of experimental reproducibility (see: Statistical Methods S1 in File S1). Further analysis of the data then ensued.

**Figure 5:**
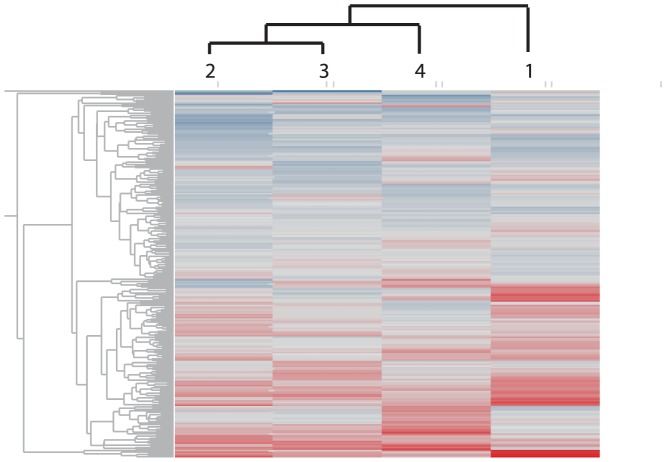
Hierarchical clustering of all data points (ΔCt) from 4 separate siRNA screen experiments outlining effects of 240 siRNA genes on loss (+ΔCt, red) or gain (−ΔCt, blue) of episomes in the absence of PA25. All genes are aligned on the y-axis (left). The columns represent experiments conducted on days 1−3 (cells maintaining HPV16) and day 4 (cells maintaining HPV31).

Initially, hierarchical clustering was employed to examine the similarity of siRNA effects on Ct values generated by Set 1 (receiving 0.1% DMSO) over the 4 different experiments (Figure 5). This information was important because it helped determine whether Experiment 4 (conducted with HPV31+ cells) should be incorporated in the analysis with the other three experiments conducted with HPV16+ cells. Hierarchical clustering revealed that the two experiments with greatest similarity were 2 and 3 (Figure 5). Experiment 4 was most similar to 2 and 3, and Experiment 1 was the least related (Figure 5). In other words, the results demonstrated that the stability profile of HPV31 episomes in a screen of 240 siRNAs fell within the range of stability profiles generated for HPV16 episomes (Figure 5). Therefore, further analyses reported here were conducted with all 4 data sets.

**Figure 6:**
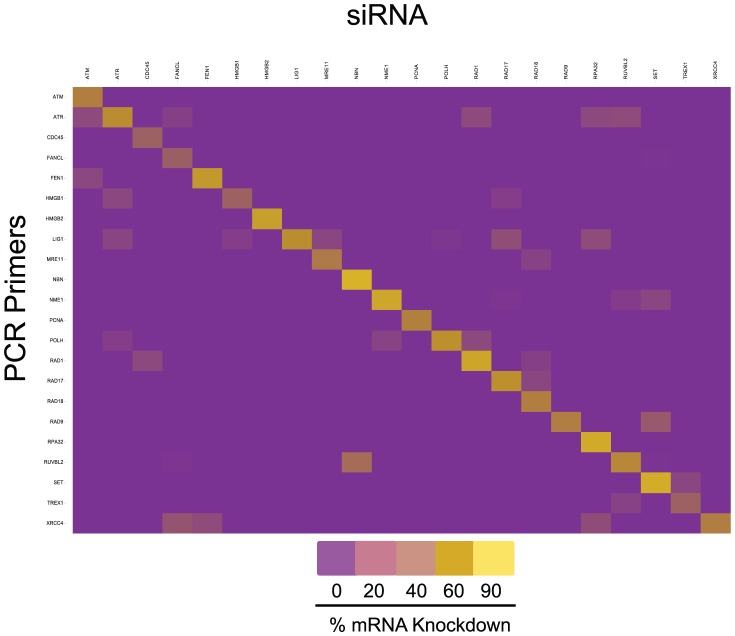
Heat map of matrix examining effects of 22 siRNAs on the expression of the same 22 genes measured by Q-PCR using gene specific primers. All 22 siRNAs were found to specifically down-regulate the appropriate target gene as seen by the mid-linear diagonal effect in the heat map.

The success of a screening experiment is dependent upon the correct siRNA pool (or compound) being arrayed in the proper well. As a test of the veracity of the manufacturer's labeling and the specificity of the siRNA pools for the targeted gene, 22 DDR siRNAs were tested in a matrix for their effects on expression of all 22 target genes by Q-RT-PCR (Figure 6). All 22 siRNAs specifically down-regulated the proper target gene in comparison to the 21 other genes in the matrix. While some slight off-target effects were noted, the resulting diagonal, mid-linear effect in the heat map indicated that the siRNAs possessed the expected specificities (Figure 6).

### Identification of DDR Genes that Control Stable HPV Episome Levels

Those siRNAs that significantly altered cell HPV episome content in the absence of PA25 were identified from the Set 1 data (Table 2); 18 genes were identified from the screen as being potentially important for HPV episome maintenance. Of these 18 genes, 7 were found to limit episome levels (knockdown resulted in increased episome numbers), while 11 genes were found to augment episome levels (knockdown caused episome loss) (Table 2). Importantly, 6 of the 18 implicated genes belonged to either homologous recombination (HR) or Fanconi Anemia (FA) pathways, which have both been previously implicated in episome maintenance. A total of 5 significant genes are involved in DSB repair including 3 associated with HR (ATM, RTEL1, RUVBL2) and 2 with non-homologous end joining (NHEJ; LIG4, POLM). Another 4 genes were members of excision repair pathways including nucleotide excision repair (NER; RAD23A), base excision repair (BER; NEIL3, RAD1), and mismatch repair (MMR; MLH3). The remaining 6 genes included TP53, UBE2V2, MTOR, and genes implicated in DNA adduct repair (TDP1, TDP2), and post-replication repair (RAD18).

**Table 2:** Genes identified in the siRNA screen that significantly alter episome levels in cells under conditions of normal maintenance.

Gene	Episomes (Fold Δ)	Activity	Repair
ATM	−3.81	PI-3 Kinase; DSB repair	HR
RTEL1	−6.95	ATP-dependent helicase; HR suppressor	HR
RUVBL2	−3.37	helicase essential for DSB repair	HR
FANCC	−4.13	Fanconi anemia pathway	FA
FANCF	2.66	Fanconi anemia pathway	FA
FAN1 (KIAA1018)	−3.62	FANC-associated exonuclease	FA
RAD23A	3.03	ubiquitin chain receptor	NER
LIG4	3.43	DNA ligase; ssDNA break repair	NHEJ
POLM	−3.57	gap-filling polymerase	NHEJ
NEIL3	2.96	DNA glycosylase	BER
RAD1	−2.81	9-1-1 complex member	BER
TDP1	5.40	Tyrosyl-DNA phosphodiesterase 1	DNA adduct repair
TDP2 (TTRAP)	−6.19	Tyrosyl-DNA phosphodiesterase 2	DNA adduct repair
RAD18	−3.85	E3 ubiquitin ligase; interacts with Rad6	PRR
UBE2V2	4.15	Lys 63 ubiquitination	error-free DNA syn.
TP53	4.50	tumor suppressor; transcriptional regulator	transcript. reg. of repair
MTOR (FRAP1)	−4.06	kinase; central regulator of cell signaling /metabolism	
MLH3	−7.49	mutL homolog	MMR

**DSB:** double-strand break; **BER:** base excision repair; **MMR:** mismatch repair; **HR:** homologous recombination; **TLS:** translesion repair; **PRR:** post-replication repair; **ICL:** interstrand cross-link; **NER:** nucleotide excision repair.

### Identification of Enhancers and Repressors of PA25 Activity

**Figure 7:**
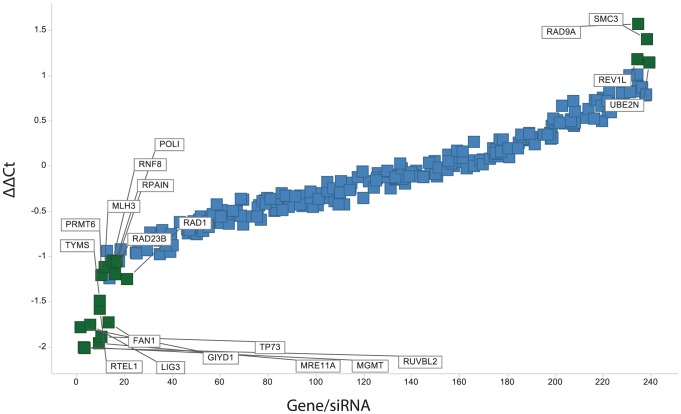
Summary scatter plot of the ΔΔCt (y-axis) arrayed from lowest to highest values. The significant genes are indicated by green. Repressors, those genes that oppose the antiviral activity of PA25, have negative ΔΔCt. Enhancers, those genes that are required for full PA25 activity, have positive ΔΔCt values.

**Figure 8:**
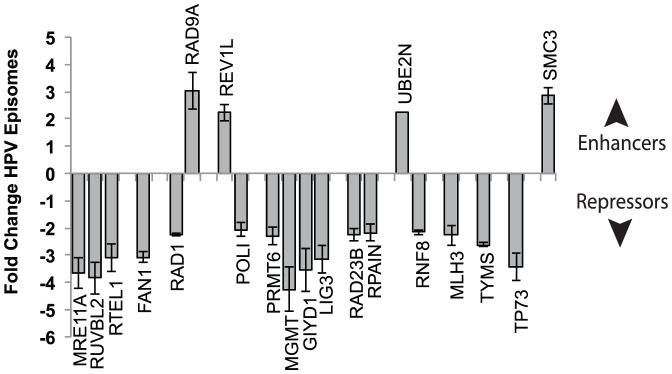
Bar graph of PA25 enhancers and repressors identified in the siRNA screen. The effects of siRNAs targeting the genes on PA25 activity are shown. Knockdown of enhancers, those genes that are required for full PA25 activity, results in a net gain in viral episomes in the presence of PA25. Knockdown of repressors, those genes that oppose the antiviral activity of PA25, causes an increase in PA25 activity resulting in a greater loss of HPV episomes.

By subtracting the Ct value for a given Set 1 siRNA from the corresponding Set 2 value (ΔΔCt, or difference of the difference), the siRNAs that significantly increased or decreased the activity of PA25 were identified (Figure 7, Table 3). A total of 21 genes were initially identified as significant in the screen. Each significant gene was then subjected to a validation test that required at least 2 of the 4 siRNAs from the original siRNA pool cause ≥2-fold change in HPV episomes in the presence of PA25. These studies confirmed 20 genes: 16 genes were designated as PA25 repressors because their knockdown resulted in increased PA25 activity (Figure 8, Table 3), while 4 genes were named PA25 enhancers since they were required for full PA25 activity.

**Table 3:** Repressors (Cause Episome Loss with Knockdown) and Enhancers (Cause Episome Gain with Knockdown) of PA25 Activity in W12E cells.

Gene	Episomes (Fold Δ)	Activity	Repair	
MRE11A	−3.67	MRN member; Endo-exonuclease	DSB, HR	Repressors
RUVBL2	−3.85	Helicase, acetyltransferase complex member	DSB, HR	
RTEL1	−3.11	Helicase; resolves DNA 2’ structures	DSB, HR	
FAN1	−3.07	FANCD2-associated nuclease	HR, ICL	
RAD1	−2.23	9-1-1 complex; Exonuclease	LP-BER	
TP73	−3.43	Transcription Factor (p53 family)	Pro-apoptotic	
POLI	−2.06	DNA Polymerase	TLS	
PRMT6	−2.30	Methyltransferase	BER	
MGMT	−4.26	Methyltransferase (alkylating agents)	BER	
GIYD1	−3.56	Structure-specific endonuclease (alkyl. agents)	Resolves HJs	
LIG3	−3.14	DNA Ligase (alkylating agents)	BER	
RAD23B	−2.25	Ubiquitin-mediated proteolytic pathway	NER	
RPAIN (MGC4189)	−2.17	RPA interacting protein	NER	
RNF8	−2.16	E3 ubiquitin-protein ligase	DSB	
MLH3	−2.28	MutL protein homolog	MMR, PRR	
TYMS	−2.62	Thymidylate synthetase		
UBE2N	2.23	E2 ubiquitin-conjugating enzyme	DSB, PRR	Enhancers
SMC3 (CSPG6)	2.85	Maintenance of chromosomes (cohesin complex)	HR	
RAD9A	3.03	Exonuclease (9-1-1 complex)	LP-BER	
REV1L	2.23	Deoxycytidyl transferase	TLS	

**DSB:** double-strand break; **LP-BER:** long-patch base excision repair; **MMR:** mismatch repair; **HR:** homologous recombination; **TLS:** translesion repair; **PRR:** post-replication repair; **ICL:** interstrand cross-link; **NER:** nucleotide excision repair; **HJs:** Holliday Junctions.

**Figure 9:**
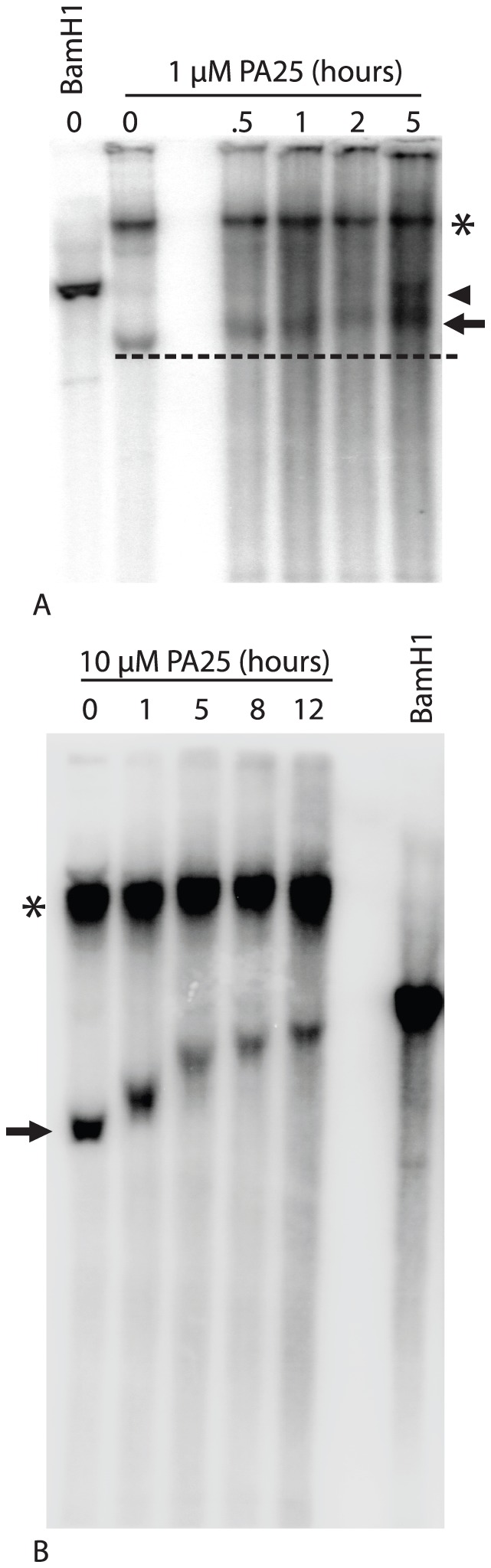
Southern blots of PA25 treated HPV16 episomes from W12E cells. A. Southern blot of intact HPV episomes following treatment over 5 µM PA25. Migration of linearized HPV16 is shown (BamH1). Note retardation of migration of HPV16 Form 1 DNA (arrow) over time in the presence of PA25, and the appearance of Form 3 viral DNA (linear) at 5 hours of treatment (arrowhead). Open circle form of HPV is indicated with asterisk. B. Southern blot of episomes following treatment with 10 µM PA25. Migration of linearized HPV16 is shown (BamH1). Note the pattern of migration of HPV16 Form 1 DNA (arrow) over time in presence of PA25 resulting in a step-like appearance of HPV topoisomers. Open circle form of HPV is indicated with asterisk.

These results suggested that PA25 might act to alter or damage episomes resulting in a DDR that either protected the viral DNA (repressors) or promoted HPV DNA loss from cells (enhancers). This hypothesis implied that prior to episome loss following PA25 treatment alterations in HPV episome structure might be detected. The earliest times following PA25 treatment were therefore examined by Southern blotting. Treatment with 1 µM PA25 for 5 hours revealed compound-dependent effects on episomal DNA (Figure 9A). The migration of the viral supercoiled form was slowed over time, and a band that co-migrated with the 8 kB linearized episome appeared at 5 hours. These results suggested that PA25 caused alterations in HPV superhelicity, and that a DSB might be generated by PA25 within a subset of viral episomes (Figure 9A). Treating with 10 µM PA25 caused a clear retardation of the HPV episome supercoiled form over time resulting in a striking, step-like pattern of topoisomers (Figure 9B).

**Figure 10:**
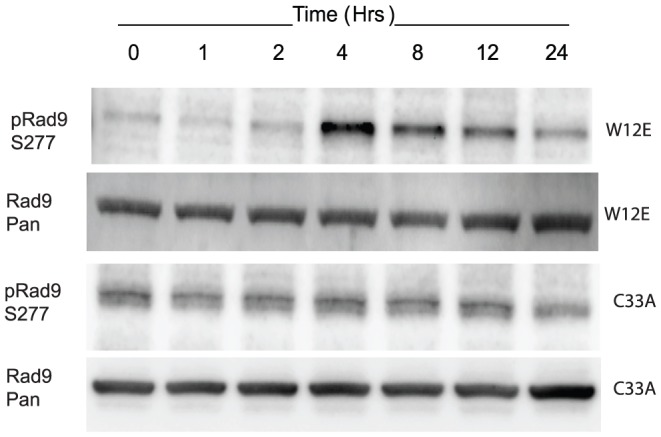
Western blot showing effects of 1 µM PA25 over time on Rad9 phosphorylation in two cervical cell lines, W12E and C33A. Phosphorylation of Rad9 (S277) peaks in the W12E samples at 4 h. after which the signal is attenuated over the remaining time course. A similar phosphorylation event is not noted in the HPV-negative C33A cells. The Rad9 (Pan) Western blots are provided as loading controls.

Rad9 and Mre11 were selected as representatives of the PA25 enhancers and repressors for further study since multiple members of their respective complexes (9-1-1 and MRN) were implicated in the gene expression and siRNA screens. Rad9 is a member of the 9-1-1 complex, which acts as a sensor of DNA damage. It was hypothesized that Rad9 might be phosphorylated in W12E cells in response to PA25 treatment. Western blotting with a phospho-specific Rad9 (S277) antibody of a 1 uM PA25 treatment time course demonstrated a sharp increase in Rad9 phosphorylation at 4 h followed by a gradual decline of signal (Figure 10). On the other hand, no such response to PA25 treatment was elicited in C33A cells, an HPV-negative cervical carcinoma cell line (Figure 10), again demonstrating the specificity of PA25 for HPV episomal DNA.

**Figure 11:**
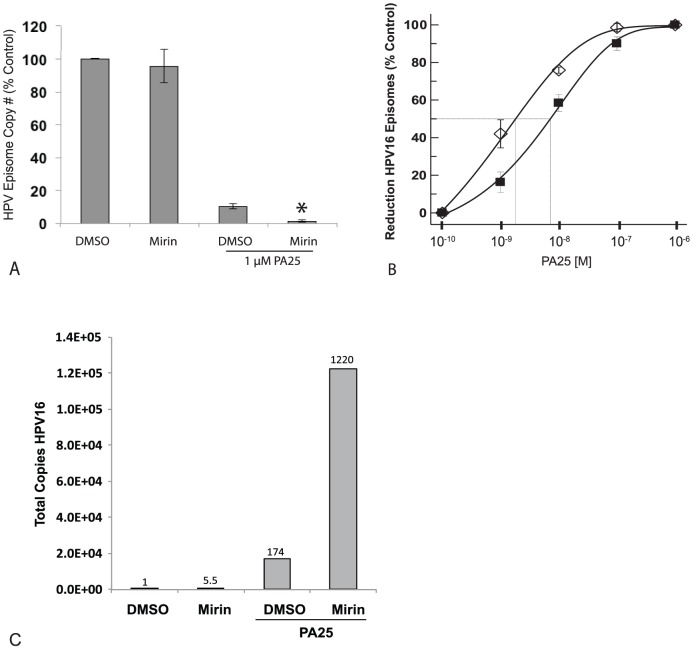
The Mre11 inhibitor Mirin acts as a PA25 sensitizer in W12E cells. A. Mirin has no effect on HPV16 episome levels by itself. PA25 causes ∼90% loss of HPV16 episomes in cells pre-treated with vehicle (0.1% DMSO), while showing a ∼98% loss of episomes in cells pre-treated with 100 µM Mirin. * p  =  0.00001 (two-tailed student’s t-test assuming unequal variance), n = 6, error bars represent standard deviation. B. Mirin (100 µM) causes a leftward shift in the PA25 dose response curve demonstrating the increased sensitivity of HPV episomes under conditions of Mre11 inhibition. The IC50 in this experiment for PA25 was 72 nM without Mirin (solid boxes), and 18 nM in the presence of Mirin (open diamonds). C. Single and double strand DNA breaks were detected by ELCQ. PA25 caused an increase in the number of detectable breaks while Mirin significantly enhanced this effect. The numbers over the bars indicate the fold change in detected HPV DNA from the vehicle (0.1% DMSO) treated control, which is set at 1.

Mre11 stood out as an intriguing PA25 repressor because its role in DNA repair makes it a plausible candidate to oppose the action of an agent that acts to cause DSBs. It was hypothesized that Mre11 inhibition would act to potentiate the action of PA25. Cells were treated with 100 µM Mirin or vehicle for 24 hours, and then treated with 1 µM PA25 (Figure 11A) for an additional 24h. Mirin treatment alone had no effect on HPV episome levels, and PA25 alone elicited a 90% episome decrease in cells, as expected (Figure 11A). Strikingly, Mirin dramatically facilitated PA25-dependent HPV episome elimination (Figure 11A) suggesting that Mre11 acts to oppose PA25 antiviral activity. A comparison of PA25 dose response curves in the presence or absence of Mirin was then conducted. Mre11 inhibition significantly sensitized HPV episomes to PA25 resulting in a leftward shift of the IC50 curve and a significant decrease in the PA25 IC50 from 72 nM to 18 nM (Figure 11B).

The ability of PA25 to directly cause single- or double-strand breaks in HPV episomal DNA was then directly tested in pull down experiments by ELCQ (Figure 11C). These experiments showed that Mirin alone resulted in approximately a 5-fold increase in HPV DNA end labeling compared with DMSO controls. On the other hand, PA25 alone resulted in a ∼170-fold increase in HPV DNA pulled down compared to vehicle-treated controls indicating that PA25 treatment causes significant DNA strand breakage within viral genomes (Figure 11C). PA25 treatment of Mirin pretreated W12E cells resulted in a ∼5-fold greater amount of end-labeled HPV DNA consistent with Mre11 inhibition sensitizing HPV genomes to PA25-dependent damage (Figure 11C).

## Discussion

The mechanism by which hairpin N-methylpyrrole-imidazole polyamides (PAs) act to destabilize and eliminate HPV episomes from cells was examined. Evidence from PCR arrays, a siRNA screen, and functional studies suggests that DDR repair pathways mediate the antiviral activity of PA25. The MRN and the Rad9-Hus1-Rad1 (9-1-1) complexes, in particular, appear to play an important role in the massive HPV episome loss promoted by PA25. The expression of Rad1, Mre11 and Nbs1 genes was altered in response to PA25 treatment. Mre11, Rad1, and Rad9 also emerged in the siRNA screen as highly significant regulators of episome stability in the presence of PA25. These data, as well as Southern blots and functional studies, suggest that HPV DNA episome alteration or damage by PA25 is a key, rate-limiting feature of its antiviral activity. PAs bind to DNA and cause a number of biophysical effects such as widening the minor groove, shrinking the major groove, and stiffening the double helix [29]. In the context of the HPV open circle or negatively super-coiled episome, PAs may cause twisting, exposure of ssDNA, or DSBs. In addition, various DNA repair pathways will recognize the bulky PAs bound to the minor groove as problematic. A broad DDR is mounted resulting in the elimination of episomes by a process that is poorly understood. It is important to note that, unlike the DDR elicited by damage to cellular genomes, the DDR elicited by PA25 does not result in robust cell cycle arrest or have large effects upon cell growth or apoptosis. We believe that this difference is attributable to both the unique niche occupied by HPV genomes within the nucleus and to the well-known action of the HPV oncogenes.

The damage to HPV episomes by PA25 is specific according to the following criteria: **1.** Inactive PAs do not trigger a DDR, **2.** PA25 activates a DDR response in HPV episome-bearing W12E cells that is qualitatively and quantitatively distinct from HPV-negative C33A cells and SiHa cells, which carry integrated copies of HPV16. These findings indicate that PA25 specifically acts on HPV in the context of the viral episome, **3.** PA25 triggers phosphorylation of Rad9 in HPV-positive W12E cells but not in C33A cells, and **4.** Pharmacological and siRNA inhibition of Mre11 potentiates PA25 antiviral activity by sensitizing HPV genomes. PA25 and other antiviral PAs do not cause measurable toxicity in HPV episome-bearing cells or HPV-negative cells indicating that these compounds do not significantly compromise cell genomes [8], [9]. These data are fully consistent with numerous reports in the literature attesting to the low toxicity of this class of compound [39], [40], [41].

It was possible to observe transient alterations in episome structure attributable to PA25 by focusing on the earliest times of treatment (Figure 9A and B). A retardation of migration of supercoiled DNA is noted at the earliest times after treatment. These changes in electrophoretic migration are attributed to structural alterations in the supercoiled episome, and not increased mass due to PA25 binding, since it is highly unlikely that PA25 survives the extensive deprotonation during the DNA isolation procedure. Also, no such alterations in electrophoretic migration occur in the matched, BamH1-linearized samples, indicating that an alternative, perhaps relaxed, structure of the HPV supercoil is responsible. In the presence of 10 µM PA25, the appearance of a series of HPV topoisomers over time was clearly observed (Figure 9B). We hypothesize that these topoisomers arise by topoisomerase resolution of PA25-generated stress within HPV supercoiled DNA. After 5 hours in the presence of 1 µM PA25, a band co-migrating with the 8 kb linearized HPV standard also appeared suggesting that dsDNA breaks are occurring within the altered supercoiled episome (Figure 9A). The data from end labeling of HPV genomes coupled to Q-PCR (ELCQ; Figure 11C) also demonstrates the generation of PA25 induced breaks within the viral genomes.

It is important to note that PAs and other minor groove-binding agents affect DNA structure in a number of quantitative and qualitative ways [29], [30], [31], [32]. Most recently, 6- and 8-ring polyamides considerably smaller than PA25 were shown to affect DNA bending in a manner dependent on DNA and polyamide (PA) sequence. The 6-ring PA bent DNA 5.4° per helical turn while 8-ring PAs bent DNA 0, 3.0 and 3.7° per turn under conditions benchmarked by straight DNA and an A_5_ sequence bent 18° per turn [42]. The stiffening of entire helical turns of DNA by PA25 could cause, for example, unequal distribution of supercoiling in an episome, resulting in unusual topologies or stretches of ssDNA that are recognized as damaged sites by the DDR. The work described here clearly shows that PA25 elicits a robust and complex DDR in cells carrying HPV episomes, but not in cells that have integrated HPV16 DNA (SiHa) or are HPV negative (C33A). DNA twisting and breaks may arise because of stress from PA25 binding within the HPV genome that is impossible to mitigate by unwinding because of its circular nature. Mre11 protects the viral genome from PA25 under these conditions.

The MRN complex is particularly interesting as a PA25 repressor. Both MRE11 and NBS1 genes are down-regulated in response to PA25 treatment, as is the MRN structural and functional partner CtIP (RBBP8), which mediates DSB resection during repair [27]. MRE11 also emerged as a strong repressor of PA25 in the siRNA screen, which led us to consider whether pharmacological inhibition of Mre11 would potentiate PA25 antiviral activity. Since Mre11 is generally regarded as both a sensor and central mediator of DSB repair [43], our data strongly argue that PA25 initiates HPV episome elimination by introducing DSBs within the HPV episome. The altered regulation of numerous other DSB repair genes by PA25 including ATM, CHEK2, RAD1, and XRCC4 is fully consistent with this idea (Table 1).

Drugs that act within DDR pathways to sensitize cells to radiation or to chemotherapeutic agents have the potential to enhance and extend cancer therapy by magnifying the toxic effect within the targeted cells [44]. These approaches work by preventing repair and allowing the accumulation of extensive DNA damage that proves toxic to the targeted cells. Mirin is an inhibitor of Mre11-associated exonuclease activity that was identified in a forward genetic screen, and subsequently shown to block the ability of MRN to repair DNA via HR [34]. We show here that Mirin makes viral episomes more susceptible to PA25 in a manner that is analogous to radiation and chemotherapeutic sensitizers. This observation was made after Mre11 emerged from the siRNA screen as a repressor of PA25 antiviral activity. These results--that PA25-dependent elimination of episomes is repressed by Mre11 and sensitized by Mirin--provides a powerful argument that PA25 acts to damage viral episomes, causing them to be eliminated from cells. It should be noted that PA25 is not toxic to cells at concentrations vastly exceeding those used in this study, and therefore the effect on episomes is a specific DNA damage event that is primarily manifested by viral DNA elimination rather than cell death [8], [9]. The lack of apoptosis genes affected by PA25 is consistent with this concept (Figure 3, Table 1).

The heterotrimeric Rad9-Hus1-Rad1 (9-1-1) complex is also found to play a role in PA25-dependent loss of HPV episomes. The 9-1-1 complex is a processivity clamp related to PCNA that acts as a scaffold to bring DDR and checkpoint effectors to sites of DNA damage [45], [46]. The 9-1-1 complex has been found to associate with and stimulate numerous checkpoint and base excision repair enzymes, and therefore may serve to coordinate DNA repair and checkpoint control [47], [48], [49]. RAD1 emerged as the gene most altered by PA25 in expression studies (Figure 3, Table 1). Rad1 and Rad9 were also both identified in the siRNA screen as highly significant, but oppositional, modulators of PA25 activity: Rad1 acts to repress PA25 activity while Rad9 is an enhancer (Figures 7 and 8, Table 3). The binding of hairpin polyamides, such as those employed in this study, is known to expand the minor groove and shrink the major groove of DNA. In contrast, association of the 9-1-1 complex with DNA has the opposite effect, resulting in a contraction of the DNA minor groove and an expansion of the major groove [46]. For these reasons the 9-1-1 complex is a good candidate sensor and coordinator of PA25 antiviral activity that may serve to recruit an enzymatic mechanism to the HPV episome resulting in its ultimate destruction. At present it is not understood why Rad1 serves as a PA25 repressor while its 9-1-1 partner Rad9 acts as an enhancer. This difference may be attributable to alterations in 9-1-1 stoichiometry following siRNA treatment, or to differences in their reported DNA binding properties [46].

The process of studying the PA25 mechanism of action required that the effects of 240 DDR siRNAs also be observed on normal HPV stability in the absence of PA25. HPV16 and HPV31 episome levels fluctuated in cells in response to a panel of DDR siRNAs in a similar manner even though the W12E cell line was derived from a cervical biopsy while the cells maintaining HPV31 were generated in a laboratory from foreskin keratinocytes. The four experiments used in these studies included three experiments (days 1−3) utilizing W12E cells and 1 experiment (day 4) using keratinocytes maintaining HPV31 episomes. A previous report indicated that these genotypes use different modes of DNA replication in cells [50]. Using hierarchical clustering (Figure 5) we established that the day 4 (HPV31) data falls within the range of similarity established for days 1−3 (HPV16). Using this approach, multiple HR and FA pathway siRNAs were identified as significant modifiers of normal HPV episome levels (Table 2). FANCC and FANCF were found to contribute to HPV episomal stability (Table 2). Since the FA and HR pathways were previously implicated in HPV persistence and the viral life cycle [9], [16], [22], [24], [28], these correlations serve as further support for the validity of our studies, and further implicate these pathways as central to the survival and persistence of HPV episomes in cells. Likewise siRNAs for TP53 and MTOR significantly affect HPV episome levels--siRNA to TP53 resulted in an increase in episomes while MTOR knockdown decreased episome levels. Both of these proteins are known targets of HPV E6, which is required for episome maintenance in cells [51], [52], [53].

A number of novel genes are also identified as highly significant in normal HPV episome maintenance by our siRNA study (Table 2). Members of excision repair, mismatch repair, and other DDR pathways are highly significant in the siRNA screen and therefore excellent candidate modifiers of episome stability (Table 2). Notably, both TDP1 and TDP2, the only known human genes whose activities are required for removing trapped DNA cleavage complexes of topoisomerase I (TDP1) and topoisomerase II (TDP2) [54], [55], were both identified as regulators of HPV episome stability. TDP1 knockdown causes a net gain of episomes while TDP2 knockdown results in a net loss (Table 2). These genes were originally identified by their ability to remove topoisomerase cleavage complexes created in the presence of topoisomerase poisons. However, there is a growing appreciation that DNA metabolic events including DNA damage, ssDNA breaks, and misinsertion of ribonucleotides can trap topoisomerase I, while transcription-related events and abasic sites may cause trapping of topoisomerase II [56]. We therefore hypothesize that the trapping of both topoisomerase I and II on HPV episomes are events that must be overcome for successful HPV episome maintenance. Together, these observations from the siRNA screen make a significant contribution to an expanding knowledge base that seeks to understand the role of DDR pathways in normal HPV episome maintenance.

It is apparent that HPV episome stability can be greatly diminished under certain conditions. Here we show a role for multiple DDR genes in mediating the loss of HPV DNA in response to PA25. It was previously shown that the ATR/Chk1 pathway does not play a role in PA25-mediated episome loss, yet does have its own role in controlling viral DNA stability [9]. However, it should be noted that it remains to be determined in both cases how viral DNA destruction is executed by the cell. We have shown here that the 9-1-1 complex is a candidate sensor that may play a role in calling for HPV episome removal, but the elements that mediate viral DNA destruction remain unknown. Likewise, Mre11 and MRN appear to play a role in stabilizing episomes against PA25-dependant damage, but the mediators of viral DNA destruction downstream of Mre11 remain to be determined. We believe that understanding the genes and processes that mediate HPV DNA instability and destruction has the potential to inform us about such diverse topics as viral persistence, interferon-mediated viral DNA loss, and antiviral therapy. Finally, it is interesting to consider the application of similar strategies to other DNA viruses that maintain their genomes as extrachromosomal plasmids.

## Supporting Information

File S1 This file contains Figure S1-Figure S4, Table S1-Table S4, and Statistical Methods S1. Figure S1, Scatter plot showing effects of PA25 on gene expression of cell cycle and DDR genes in the HPV-negative C33A cervical cancer cell line and in the HPV16 positive SiHa cervical cancer cell line. A total of 7 genes were found to have altered expression in both cell types with only a single gene difference between both. Also see Table S1 in File S1. Note similarity between the two graphs for comparison to Fig. 3B and Table 1. Figure S2, FACS analysis was conducted on cells treated with vehicle (control) or with 10 µM PA25 for 48 h. Values indicated are the % Total cells within the relevant cell cycle period. Figure S3, Control data showing the resulting control mean and SD’s from each of the experimental days from siRNA screen. Figure S4, The coefficient of variation values are well below 5%, and fairly consistent across all experimental days. Table S1, Summary of gene expression changes in HPV-negative (C33A) and HPV-positive (SiHa) cells following treatment with 10 µM PA25. Table S2, CVs for Each Experimental Day. Table S3, All 240 Genes in Dharmacon DDR siRNA Library. Table S4, PCR primer sequences for 22 DDR genes employed in matrix for verification of siRNA specificity. Statistical Methods S1, Control Ct values (Step 1) and Experimental Ct values (Step 2).
(DOCX)
