# Supplementary material for: DNA Damage Repair Genes Controlling Human Papillomavirus (HPV) Episome Levels under Conditions of Stability and Extreme Instability
Source: PLoS One. 2013 Oct 2;8(10):e75406. doi: 10.1371/journal.pone.0075406 (PMC3788802; doi:10.1371/journal.pone.0075406)
Supplement: File S1 — This file contains Figure S1-Figure S4, Table S1-Table S4, and Statistical Methods S1. Figure S1, Scatter plot showing effects of PA25 on gene expression of cell cycle and DDR genes in the HPV-negative C33A cervical cancer cell line and in the HPV16 positive SiHa cervical cancer cell line. A total of 7 genes were found to have altered expression in both cell types with only a single gene difference between both. Also see Table S1 in File S1. Note similarity between the two graphs for comparison to Fig. 3B and Table 1. Figure S2, FACS analysis was conducted on cells treated with vehicle (control) or with 10 µM PA25 for 48 h. Values indicated are the % Total cells within the relevant cell cycle period. Figure S3, Control data showing the resulting control mean and SD’s from each of the experimental days from siRNA screen. Figure S4, The coefficient of variation values are well below 5%, and fairly consistent across all experimental days. Table S1, Summary of gene expression changes in HPV-negative (C33A) and HPV-positive (SiHa) cells following treatment with 10 µM PA25. Table S2, CVs for Each Experimental Day. Table S3, All 240 Genes in Dharmacon DDR siRNA Library. Table S4, PCR primer sequences for 22 DDR genes employed in matrix for verification of siRNA specificity. Statistical Methods S1, Control Ct values (Step 1) and Experimental Ct values (Step 2). (DOCX) [file pone.0075406.s001.docx]

**Figure S1:**

| **Table S1**: Summary of gene expression changes in HPV-negative (C33A) and HPV-positive (SiHa) cells following treatment with 10µM PA25 | | | | |
| --- | --- | --- | --- | --- |
| Gene | C33A Gene Expression (Fold Change) | SiHa Gene Expression  (Fold Change) | Activity | Repair |
| ERCC3 | -36.76 | -40.22 | Helicase; subunit of TFIIH | NER |
| ERCC4 | -6.45 | -4.11 | Structure-specific DNA repair endonuclease | HR, NER |
| ERCC5 | No change | -3.41 | Structure-specific DNA repair endonuclease | NER |
| XPA | -22.01 | -54.95 | Xeroderma pigmentosum group A-complementing protein | NER |
| XRCC4 | -8.00 | -9.32 | X-ray repair cross-complementing protein | NHEJ, DSB |
| LIG3 | -2.73 | No change | DNA ligase (alkylating agents) | BER |
| RAD51L1 | -6.23 | No change | Recombination repair protein | HR, DSB |
| RAD51C | No change | -2.38 | Recombination repair protein | HR |
| RAD51L3 | -7.67 | -8.06 | Recombination repair protein | HR |

**Figure S2**

**Statistical Methods**

**Control Ct values (Step 1).** On each experimental day six (6) controls were run. A pooled estimate of the standard deviation was calculated using the analysis of variance. A studentized residual was then calculated. Any control value that expressed a studentized residual > |2| was classified as an outlier and removed from further calculations. Fig. S1 displays the resulting control mean and SD’s from each of the experimental days.

**Experimental Ct values** **(Step 2).** For each gene, Ct values were declared as outliers if they had values that were well beyond a reasonable limit (i.e., Ct of 40). For those rare cases, the Ct values were adjusted to the average of the remaining 3 values. Genes were declared significant if the difference from the average control value was > |1.0|. That difference was used since it translates to 2-fold change. Note that the average SD was observed to be 0.49 for the controls in Set 2. When a z-score is calculated as (Ct value – control mean) / SD, using the 2-fold change for significance just described, that translates to 1 / 0.49 = 2.04, which is also significant using the z-score criteria. Therefore, the 2-fold change criterion is equivalent to the statistical z-score criteria for selecting genes that differ from the control values.

**Figure S3**


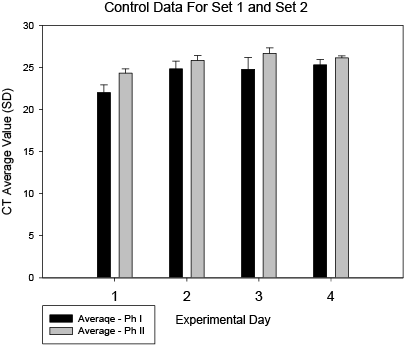


**Graphical Activity Display (Scatter Plot).** Displays values for genes that satisfy the following criteria: If they are declared significant using the rule stated above (Step 2) for both Set 1 and Set 2 then [(Set 1 Ct – control mean Ct for Set 1) – (Set 2 Ct – control mean Ct for Set 2)] is calculated and displayed. This is the typical difference of differences value (ΔΔCt).

If the (Set 1 Ct – control mean Ct for Set 1) is not declared significant using Step 2, then the Set 1 difference is declared to be 0.0 in the difference of difference calculation. This allowed us to look for additive or synergistic effects between siRNA treatment alone versus siRNA plus PA25 treatment on HPV episome stability.

**Clustering.** Hierarchical agglomerative clustering was used to generate the heat map (Spotfire, TIBCO Software). For row (column) clustering, the cluster analysis begins with each row (column) placed in a separate cluster. Then the distance between all possible combinations of two rows (columns) is calculated using a Euclidean distance measure. The two most similar clusters are then grouped together and form a new cluster. In subsequent steps, the distance between the new cluster and all remaining clusters is recalculated using the UPGMA method (Sneath and Sokal, 1973).

**Experimental Quality Assessment.** Since there were no positive controls in this experiment, direct calculation of popular measures such as the z-factor (Zhang, et. al. 1999) that provide an assessment of data quality was not possible. Instead of the z-factor, calculation of the coefficient of variation (CV) was performed for each experimental day for the control data.  Those summaries may be found in Table S2.

**Table S2:** CVs for Each Experimental Day

| Experimental Day | Set 1 CV (%) | Set 2 CV (%) |
| --- | --- | --- |
| 1 | 3.864093328 | 4.282085398 |
| 2 | 3.532568762 | 1.708431985 |
| 3 | 2.593454208 | 3.466556865 |
| 4 | 2.06298361 | 0.95912775 |

The CV’s are well below 5%, and fairly consistent across all experimental days. This is displayed in Fig. S4.

**Figure S4:**


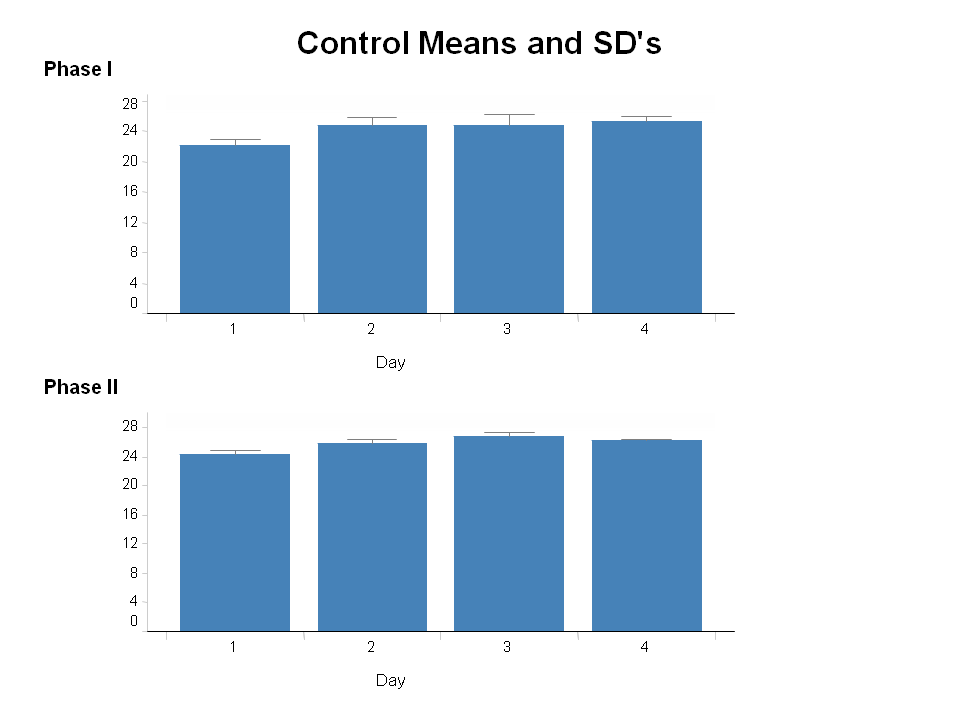


**References:**

Sneath PHA and Sokal RR (1973) Numerical Taxonomy (pp; 230-234), W.H. Freeman and Company, San Francisco, California, USA

Zhang J, Chung TY, and Oldenburg KR (1999) A simple statistical parameter

for use in evalution and validation of high throughput screeing assays.

J. Biomol. Screening 4: 67–73.

**Table S3:** All 240 Genes in Dharmacon DDR siRNA Library

| RAD50 | POLK | ADPRTL3 | POLA | PMS1 | C7ORF11 | CHEK2 | FANCA |
| --- | --- | --- | --- | --- | --- | --- | --- |
| POLE2 | SHFM1 | NEIL2 | RAD9A | BRCA1 | HMGB1 | NUDT1 | KIAA0625 |
| RUVBL2 | NEIL3 | REV1L | RENT1 | POLM | RAD54B | MBD4 | FLJ10719 |
| PRKCG | UBE2A | SOD1 | NBS1 | REV3L | ERCC6 | RNF168 | UBE2V2 |
| FANCC | HRMT1L6 | CSNK1D | DMC1 | HMGB2 | LIG1 | PRPF19 | SMUG1 |
| FEN1 | RNF8 | MSH3 | PCNA | GADD45A | RPA3 | FRAP1 | RAD21 |
| TCEA1 | TP53 | MSH4 | BAZ1B | IGHMBP2 | CHAF1A | RAD23B | RAD51L1 |
| RTEL1 | RPA2 | XAB2 | ALKBH | PMS2 | SPO11 | MJD | UNG |
| GCN5L2 | MMS19L | FANCG | POLB | CSNK1E | DNMT1 | DCLRE1C | CHEK1 |
| APTX | MGC2731 | ATR | NTHL1 | BRIP1 | USP1 | FANCB | ATRIP |
| RAD18 | POLN | HEL308 | DDB2 | CSPG6 | EYA1 | CETN2 | DUT |
| TTRAP | MIZF | RAD51L3 | POLD1 | RAD52 | RECQL4 | KUB3 | PNKP |
| GTF2H5 | MSH6 | UNG2 | MGMT | FANCL | RAD52B | TP73 | BLM |
| POLE | FANCE | GTF2H2 | FANCF | FANCD2 | MLH3 | OGG1 | APEX2 |
| UBE2B | EME2 | YBX1 | PARG | TRIP13 | CIB1 | LIG3 | BRCA2 |
| MDC1 | C2ORF13 | XRCC1 | ERCC2 | TYMS | BTG2 | MEN1 | G22P1 |
| IHPK3 | TP53BP1 | GTF2H1 | TADA3L | XPC | MPG | MLH1 | CLK2 |
| SIRT1 | MNAT1 | ERCC5 | ATRX | HUS1 | TNP1 | MRE11A | POLG |
| TREX1 | PMS2L5 | MUS81 | UBE2V1 | RPS27L | MSH2 | RRM2B | BRE |
| WRN | SMC6L1 | RAP80 | POLL | DNA2L | RAD51 | FLJ40869 | XRCC5 |
| DDX11 | CCNH | FLJ13614 | GTF2H3 | MAD2L2 | RAD1 | DCLRE1B | HSU24186 |
| APEX1 | RBBP8 | TRIM28 | EME1 | KIAA1596 | FLJ21816 | ERCC3 | XRCC3 |
| TDG | XRCC2 | POLS | POLQ | SETMAR | KIAA1018 | GIYD1 | NPM1 |
| TOPBP1 | RECQL5 | CXORF53 | RAD51C | PRKDC | CNOT7 | MUTYH | ASF1A |
| RAD54L | NEIL1 | POLG2 | MSH5 | C11ORF13 | CDKN2D | TDP1 | H2AFX |
| RPA1 | FLJ12610 | DCLRE1A | DEPC-1 | PARP2 | DDB1 | POLH | FLJ22833 |
| ATF2 | XRCC4 | UVRAG | LIG4 | POLI | CKN1 | GADD45G | UBE2N |
| VCP | DLG7 | TREX2 | ATM | RAD17 | PARP1 | EYA3 | ERCC4 |
| ALKBH2 | EXO1 | HTATIP | SMC1L1 | TOP2A | MGC32020 | XPA | ERCC1 |
| GTF2H4 | ABL1 | RECQL | CDK7 | PER1 | MGC4189 | RAD23A | RRM2 |

**Table S4:** PCR primer sequences for 22 DDR genes employed in matrix for verification of siRNA specificity.

| **Gene** | **Forward** | **Reverse** | **RefSeq #** |
| --- | --- | --- | --- |
| MRE11A | CATAGCCTCACGGACTTCATC | TCATATTGATGCCCTCGAAGAC | NM_005590 |
| RAD9A | CAGCGTCACTTCAGCCA | GAAGACTCACAACCTGTCCTT | NM_004584 |
| RAD17 | CAATCTTCCAAAGTGTCGCTTC | TCCTGACTTCTGCCTACCA | NM_133338 |
| RAD1 | GTATCTGTTGACTTGGGTCTGA | GTGAAGTCCTACAAATTACCATGTC | NM_002853 |
| RAD18 | GGTTCAATCTCAGCACTTTCAG | GCAATAGTTCCAGTTCAGACATC | NM_020165 |
| FEN1 | AGTAGCTCTTGATGTCATTCTCC | CCCGAACCAAGCTTTAGCC | NM_004111 |
| NBN | GAGACTCTTCTTTTGCATGTTGA | CACACATCATTGGAGGATCAGA | NM_002485 |
| PCNA | AGCATCTCCAATATGGCTGAG | ATGAAGTTGATGGATTTAGATGTTGA | NM_002592 |
| ATM | CCTCAACACTTCTGACCATCT | GTGCCTAAACAAAGCTCTCAG | NM_000051 |
| HMGB1 | GGATCTCCTTTGCCCATGT | CAGCCATTGCAGTACATTGAG | NM_002128 |
| HMGB2 | TCCTCATCTTCTGGTTCGTTC | CATATGAACAGAAAGCAGCTAAGC | NM_001130688 |
| LIG1 | TGAGGTAGATGAGGTCGAAGG | AGATCCAGCCATTCCAAGTG | NM_000234 |
| TREX1 | CTCTCACCACCATCCGTTC | CTGCCTGTCACTGGTATGATG | NM_016381 |
| CDC45 | CACCACAGCATGTCATTCAG | CATCCTCTTTGACTACGAGCAG | NM_001178010 |
| XRCC4 | CTCATTCAACACCAGAATAAACCG | AGGCTTCTGAGAGATTGGAATG | NM_022550 |
| RUVBL2 | CATGCCTTGCGAAGCCT | GTTACAGCCACAACCAAAGTC | NM_006666 |
| FANCL | GGAGGTAGTGCATACAGCTC | AGTACAACAGAGAATGCAGCA | NM_001114636 |
| NME1 | CGTGTAATCTACCAGTTCCTCAG | TCCGTGGAGACTTCTGCATA | NM_198175 |
| SET | CAGTAAACCAGGTAAAGAAGCTCT | GAAGTCCACCGAAATCAAATGG | NM_003011 |
| POLH | CAATCACAGCAAAACGAGACAT | CAGATCTTCTACTGGCACAAGT | NM_006502 |
| RPA32 | GGCATGATCTTAAAGGCTACCA | GACACAGATGACACCAGCA | NM_002946 |
| ATR | CCCAGACAAGCATGATCCAG | GAAGATGATGACCACACTGAGA | NM_001184 |
